# MRPL48 is a novel prognostic and predictive biomarker of hepatocellular carcinoma

**DOI:** 10.1186/s40001-023-01571-z

**Published:** 2023-12-14

**Authors:** Yu-Xiang Lin, Jun-Yong Pan, Wen-Du Feng, Tian-Cong Huang, Cheng-Zong Li

**Affiliations:** 1 Department of Hepatobiliary and Pancreatic Surgery, The Second Affiliated Hospital of Fujian Medical University (Donghai District), Quanzhou, 36200 People’s Republic of China; 2 Central Supply Service Department, The Second Affiliated Hospital of Fujian Medical University, Quanzhou, 36200 People’s Republic of China

**Keywords:** Hepatocellular carcinoma, MRPL48, Prognostic value, Biomarker, Biological functions

## Abstract

**Background:**

Hepatocellular carcinoma (HCC) is one of the most prevalent forms of cancer and poses a threat to the health and survival of humans. Mitochondrial ribosomal protein L48 (MRPL48) belongs to the mitochondrial ribosomal protein family, which participates in energy production. Studies have shown that MRPL48 can predict osteosarcoma incidence and prognosis, as well as promotes colorectal cancer progression. However, the role of MRPL48 in HCC remains unknown.

**Methods:**

TCGA, GEO, HCCDB, CPTAC, SMART, UALCAN, Kaplan–Meier plotter, cBioPortal, and MethSurv were performed for bioinformatics purposes. Quantitative RT-PCR, immunoblotting, and functional studies were conducted to validate the methodology in vitro.

**Results:**

MRPL48 was greatly overexpressed in HCC tissues, compared with healthy tissue, which was subsequently demonstrated in vitro as well. The survival and regression analyses showed that MRPL48 expression is of significant clinical prognostic value in HCC. The ROC curve and nomogram analysis indicated that MRPL48 is a powerful predictor of HCC. MRPL48 methylation was adversely associated with the expression of MRPL48, and patients with a low level of methylation had poorer overall survival than those with a high level of methylation. GSEA showed that the expression of the MRPL48 was correlated with Resolution of Sister Chromatid Cohesion, Mitotic Prometaphase, Retinoblastoma Gene in Cancer, RHO Gtpases Activate Formins, Mitotic Metaphase and Anaphase, and Cell Cycle Checkpoints. An analysis of immune cell infiltration showed a significant association between MRPL48 and immune cell infiltration subsets, which impacted the survival of HCC patients. Additionally, MRPL48 knockdown reduced HCC cell proliferation, migration, and invasion in vitro.

**Conclusions:**

We demonstrated that MRPL48 expression may be associated with HCC development and prognosis. These findings may open up new research directions and opportunities for the development of HCC treatments.

## Introduction

Hepatocellular carcinoma (HCC) is the most common primary liver cancer worldwide and poses a serious public health concern. It is estimated that 905,677 new cases of hepatocellular carcinoma were diagnosed in 2020, and 830,180 died from the disease [1]. HCC has become the sixth most common malignancy and also the third leading cause of cancer-related deaths worldwide [2]. There is no doubt that surgical treatment of HCC is crucial for long-term survival. However, because HCC develops insidiously, over 80% of patients with HCC are first diagnosed at the late stages and have missed the opportunity to receive radical surgery to cure the disease [3]. In addition, the recurrence rate of HCC is high, with a 70% rate of recurrence within 5 years [4]. There are still limited clinical options available for delaying or prolonging tumor progression, despite research into the biological and environmental mechanisms that underlie liver cancer occurrence and progression. Therefore, there is an urgent need to identify predictive biomarkers of diagnosis and treatment.

Several studies have demonstrated that mitochondria play a key role in apoptosis, metabolism and that their dysfunction may contribute to cancer development [5, 6]. The mitochondrial DNA (mtDNA) of a human cell is 16,569 bp long and contains 37 genes and codes for 13 proteins involved in cell energy metabolism [7, 8]. Due to the ability of mitochondria to adapt quickly to environmental cues, they are one of the main mediators of tumorigenesis. During the last two decades, numerous mtDNA mutations have been reported in cancers of various types, including renal adenocarcinomas, colon cancers, head and neck tumors, and ovarian cancers [9, 10]. Throughout the mitochondrial matrix, mitochondrial ribosomes translate 13 mtDNA-encoded proteins, all of which are mitochondrial respiratory chain enzymes. The oxidative phosphorylation (OXPHOS) system is mediated by mitochondrial ribosomes, which synthesize mtDNA-encoded subunits. Research has shown that mitochondrial ribosomal proteins (MRPS), which are composed of small and large subunits, play a leading role in these processes [11]. MRPS abnormal expression may be one of the factors involved in mitochondrial dysfunction, which then causes mitochondrial disease and even cancer. In ovarian cancer, a total of 10 MRPLs and 11 MRPSs showed significant prognostic significance, with MRPL49 proving to be the most predictive [12, 13]. In breast cancer, downregulating MRPS23 expression can reduce cell proliferation and increase apoptosis [14], while the overexpression of both MRPL13 and MRPS30 are associated with poorer survival [15, 16]. The MRPS have also been reported to be active in HCC, and it has been shown that high levels of MRPL13 promote the invasion of liver cancer cells [17]. In summary, the MRPS may play a crucial role in the progression of tumors.

MRPL48 belongs to the MRPS family, which is involved in the formation of ATP and fuels the growth of cells. Studies have shown that MRPL48 is of predictive value for the occurrence and prognosis of osteosarcoma, as well as promotes the growth of colorectal cancer cells [18, 19]. However, MRPL48 is not yet well understood in terms of its prognosis and regulatory mechanisms in HCC. Furthermore, immunological analysis of the tumor microenvironment appears to indicate the prognosis of HCC patients, as well as evidence of the benefit of immunotherapy. In contrast, little research has been conducted to demonstrate a link between MRPS and immune infiltration patterns.

In this study, we first evaluated the prognostic significance of the expression of MRPL48 mRNA and methylation in patients with HCC based on data from the Cancer Genome Atlas (TCGA) and Gene Expression Omnibus (GEO). Secondly, survival predictions based on the expression of MRPL48 mRNA in HCC were validated using the ICGC LIRI-JP datasets (HCCDB18). In addition, we performed GSEA analysis to further understand the biological involvement of MRPL48 in HCC pathogenesis. Finally, we conducted in vitro functional experiments using cells with MRPL48 knockdown and evaluated MRPL48 expression in HCC cell lines. In summary, the findings of this study provide insights into the clinical significance, potential functions, interactive network, and association of MRPL48 with immune infiltration in HCC, providing a novel prognostic biomarker for predicting the survival and targeting targeted treatment of HCC during its early stages.

## Materials and methods

### Repository of data

Liver hepatocellular carcinoma (LIHC) gene expression profiles were obtained from The Cancer Genome Atlas (TCGA), at level 3 of expression (level 3 data) (https://cancergenome.nih.go). This study included 374 samples from the LIHC tissues and 50 samples from paracancerous tissue (Workflow Type: HTSeq-FPKM). Then, we converted the HTSeq-FPKM values into transcript per million (TPM) values to compare the differential expression among samples. The TCGA data portal was also used to obtain relevant clinical information of the HCC patients. For the pan-cancer analysis, normal RNASeq data were collected from 33 types of tumors derived from TCGA and Genotype-Tissue Expression (GTEx) samples using UCSC Xena (https://xenabrowser.net/). Meanwhile, this study relied on R to obtain MRPL48 mRNA expression and clinical data from GSE121248, GSE164760, and GSE17967 data in the GEO databases.

### Differentially expressed genes (DEGs)

The data were extracted from TCGA-LIHC and divided into high and low MRPL48 expression groups, and the original counts matrix was differently analyzed using the DESeq2 [1.36.0] package [20]. The threshold of DEGs were determined using a | log2-fold change|> 1.0 and adjusted *p* < 0.05. The differential analysis results were visualized using the ggplot2 package in R (version 4.2.1).

### Enrichment analysis of gene ontology (GO)

MRPL48 and its relatives DEGs (|log2-fold change|> 1.5 and adjusted *P* < 0.01) were enriched to obtain GO annotations. GO enrichment analysis was conducted using the clusterProfiler (4.4.4) package in R (version 4.2.1) [21].

### Statistical analysis of gene set enrichment

For the Gene Set Enrichment Analysis (GSEA), an analytical method was used to determine whether the two phenotypes were statistically different in the presence of a previously defined set of genes. This study used the R package (version 4.2.1), clusterProfiler (4.4.4), for GSEA [21]. The purpose of this study was to determine whether the high and low MRPL48 expression groups displayed significant differences in function and pathways. To perform each analysis, 500 gene set permutations were performed. A phenotyping label was established by measuring the MRPL48 mRNA expression levels. Reference genes were chosen from the MSigDB Collections c2.cp.all.v2022.1.Hs.symbols.gmt [All Canonical Pathways]. Significant enrichment was defined as an adjusted p value of 0.05, a false discovery rate (FDR) value of 0.25, and a normalized enrichment score (NES) of more than 1.

### MRPL48 expression is correlated with immune infiltration

By integrating genes in the published signature gene lists [22], we quantified the relative tumor infiltration levels of immune cell types using the ssGSEA (single-sample Gene Set Enrichment Analysis) method in the GSVA package (1.46.0) [23]. We applied the Wilcoxon rank-sum test to examine the abundance of immune cells in different MRPL48 mRNA expression groups. A correlation between immune cell infiltration and MRPL48 mRNA expression levels was determined in TCGA HCC samples.

### Methylation levels and prognoses based on MRPL48 expression

To obtain MRPL48 copy number variation (CNV) and methylation level data, we accessed the web platform, cBioPortal (https://www.cbioportal.org/). We further examined MRPL48 gene expression variation between MRPL48 copy number variation groups (Kruskal–Wallis test) and the relationship between MRPL48 methylation level and MRPL48 gene expression (Person correlation). In addition, we analyzed and compared MRPL48 methylation levels in pan-cancer tissues and normal tissues using the SMART web platform (http://www.bioinfo-zs.com/smartapp/). At the same time, we used the UALCAN online tool (http://ualcan.path. uab.edu/) to analyze TCGA data on MRPL48 promoter methylation level differences between HCC and normal tissues. Finally, we analyzed HCC methylation status (TCGA data) using MethSurv online tool (https://biit.cs.ut.ee/methsurv/) to determine its prognostic value.

### Generating and predicting predictive models

Firstly, Akaike's information criterion (AIC) method and multivariate Cox regression analysis were used to determine the optimal prognostic model. Secondly, the R software package, rms, was used to construct a nomogram to predict prognosis. Meanwhile, based on median risk scores, patients were categorized into high- and low-risk groups. The Kaplan–Meier and a two-sided log-rank test were used to determine the OS differences between the two groups. Finally, receiver operating characteristic (ROC) curves were constructed to calculate the prediction accuracy of the prognostic model intensity.

### Culturing and transfecting cells

Immortalized liver (MIHA) and human HCC (Hep-G2, SNU-387, and Huh-7) cell lines were obtained from the American Type Culture Collection (ATCC) and China Cell Bank. The cells were cultured in DMEM medium (Gibco, Grand Island, USA) containing 10% fetal bovine serum (Gibco, Grand Island, USA). The cells were cultured in an incubator with 5% carbon dioxide at 37 °C. MRPL48 siRNA sequences (5′-GCAACTCTCTCTCCATTAAAG-3′and 5′-ACTTCAAGGGACGATTCAAAG-3′). Cells with MRPL48 knockdown were collected 72 h after transfection.

### A quantitative real-time PCR approach based on RNA extraction

Cells from human HCC cell lines were lysed in TRIzol reagent (Life Technologies, USA) to obtain total RNA. A RevertAid TM First Strand cDNA Synthesis Kit (Life Technologies, USA) was used to reverse transcribe the RNA. The following primers were used: MRPL48 forward 5′-TCGGTTTGCACAGCTAGAGG-3′ and reverse 5′-GGCACAGCACCTTTTCCAAG-3′. An internal control gene, GAPDH, was used to normalize transcriptional levels.

### Western blotting analysis

The protein concentrations of the lysed cells were measured using bicinchoninic acid assay. The cells were washed with an ice-cold PBS solution and lysed in a 100 mL RIPA buffer containing 100 mM PMSF on ice. The protein lysates were further separated on 10% polyacrylamide gels (Invitrogen), and then transferred onto PVDF (polyvinylidene fluoride) membranes. A 5% skim milk solution in PBS containing 0.1% Tween 20 was used to block the membranes for 2.5 h. Then, the membranes were incubated overnight with antibodies targeting anti-b-Tubulin (1:1000, Proteintech, Chicago, IL, USA) and anti-MRPL48 (1:1000, Proteintech). Protein–antibody complexes were detected using chemiluminescence (Life Technologies, USA) and recorded on Hyperfine-ECI detection films after conjugating with the corresponding HRP-coupled secondary antibodies.

### Cell proliferation, invasion, and migration assays

According to the manufacturer's instructions, a CCK-8 kit (Cell Signal Technology, New York, USA) was used for cell proliferation testing. And 5 × 10^3^ cells were inoculated into each well of a 96-well plate, and 200 μL of fresh culture medium containing 10 μL of CCK-8 reagent was added into them at 24, 48, 36, and 72 h after transfection, and incubated at 37 °C for 4 h. OD was measured on a 450 nm tablet reader (Bio Rad, Harkles, CA, USA). In the colony formation assay, 3000 cancer cells were inoculated into a 6-well plate and the culture medium was changed every other day. The colonies were immobilized with formaldehyde and stained with crystal violet. The cell migration ability was investigated using wound healing assays. The wounds were made using a 200 mL pipette tip at 90 percent confluency of the transfected cells. After incubation in serum-free medium for 72 h, the migration distance of the cells was calculated.

### Statistical analysis

To analyze statistical differences, *t*-tests or one-way ANOVA were performed. Kaplan–Meier analysis was used to assess the survival rate of the patients. Moreover, the log-rank test was used to evaluate survival differences. MRPL48 expression level and other clinical parameters associated with OS and DSS in patients with HCC were evaluated via univariate and multivariate Cox analyses to evaluate their independent prognostic significance. ROC curves (AUC) were established to evaluate the diagnostic significance of MRPL48 expression using the pROC package in R software. Generally, AUCs > 0.7 indicate good accuracy, while AUCs 0.5–0.7 indicate weak accuracy. We set up a nomogram to predict the OS of HCC patients based on MRPL48 expression and other clinical parameters. A p value of 0.05 was considered statistically significant. Means ± standard deviations were presented for continuous data.

## Results

### MRPL48 expression in pan-cancer samples

**Fig. 1:**
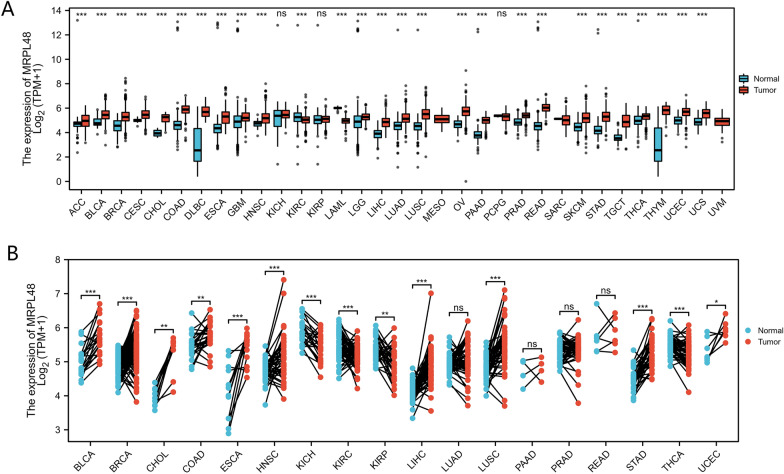
The levels of MRPL48 mRNA expression in different cancers in humans. **A** MRPL48 mRNA expression in 33 cancer types (TCGA and GTEx). **B** Expression of MRPL48 mRNA in tumors and adjacent tissues of the same patient (TCGA and GTEx). *p < 0.05, **p < 0.01, ***p < 0.001. The “ns” indicates that the data are not significant

Through the use of independent datasets from different sources, we examined the expression levels of MRPL48 in different types of cancer. MRPL48 transcriptional levels in various human cancers and in their corresponding normal tissues were investigated using TCGA and GTEx datasets. The expression of MRPL48 was significantly higher in tumor tissues than in adjacent normal tissues for almost all tumor types, including breast invasive carcinomas (BRCA) and cholangiocarcinomas (CHOLs). The results of the paired specimens also showed similar results (Fig. 1A, B).

### Data from different databases to analyze MRPL48 expression in HCC

**Fig. 2:**
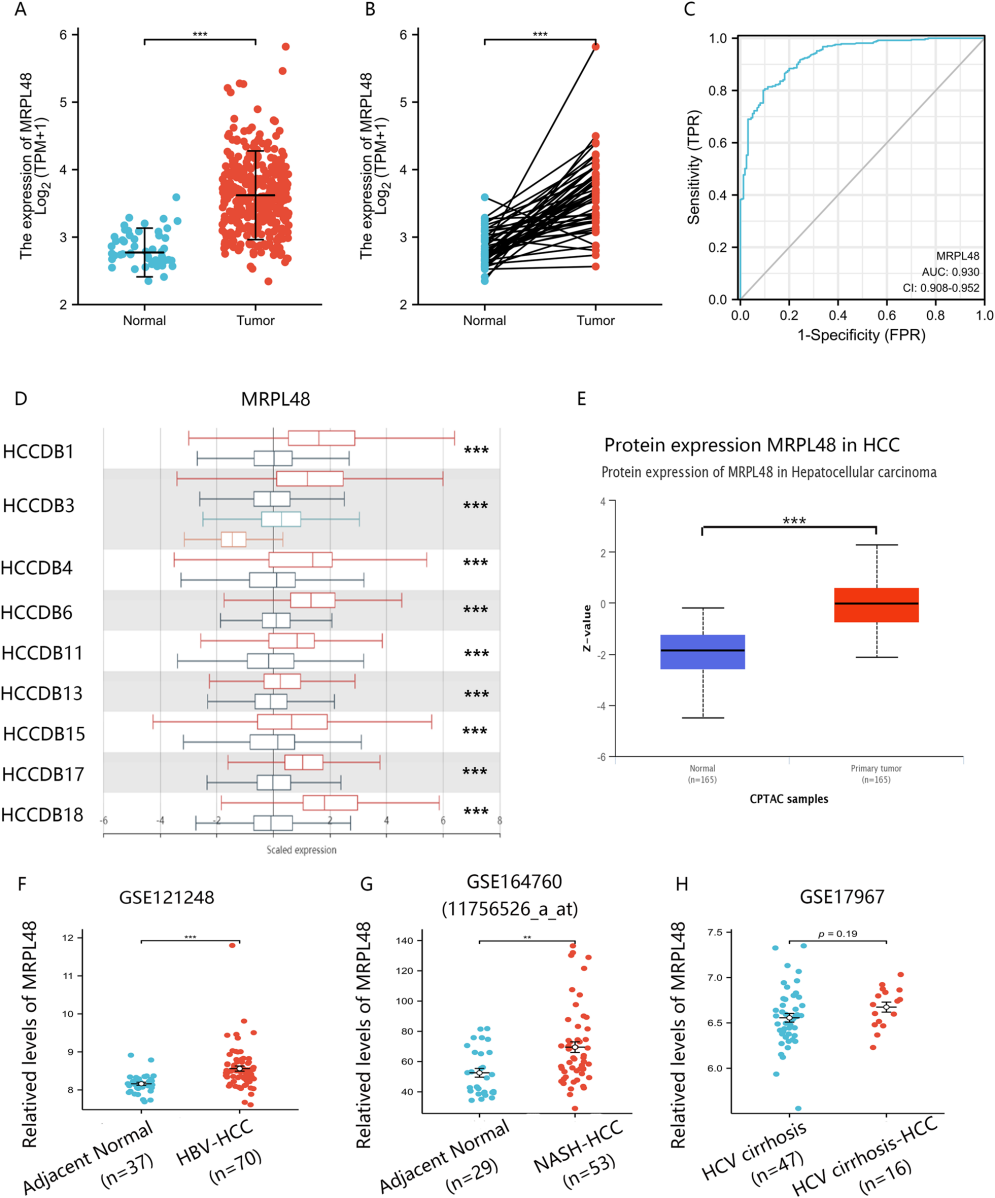
MRPL48 expression in cells and tissues of HCC. **A** The mRNA expression of MRPL48 in the tissues of normal and tumorous individuals (TCGA and GTEx). **B** The mRNA expression of MRPL48 in HCC paired tissues (TCGA and GTEx). **C** Receiver operating characteristic analysis (ROC) of MRPL48 in the context of HCC. **D** MRPL48 mRNA expression in several HCC databases (HCCDB). **E** The mRNA expression of MRPL48 in HBV-HCCs and adjacent non-tumor HBV liver samples (GSE121248). **F** The mRNA expression of MRPL48 in NASH-HCCs and adjacent non-tumor NASH liver samples (GSE164760). **G** The mRNA expression of MRPL48 in HCV cirrhosis-HCCs and HCV cirrhosis liver samples (GSE17967). Data are presented in the following format: means ± SD. *p < 0.05,**p < 0.01, ***p < 0.001

Although MRPL48 has been increasingly recognized as a new tumor biomarker, transcriptional analysis of MRPL48 in human HCC has not been extensively conducted. Firstly, we used TCGA data to measure MRPL48 transcription levels between HCC and normal samples. It was found that the levels of MRPL48 mRNA expression were significantly higher in HCC specimens than in normal liver specimens (*p* < 0.001) (Fig. 2A). A similar conclusion was also reached in paired normal tissues and HCC tissues (*p* < 0.001) (Fig. 2B). As shown in Fig. 2C, the ROC analysis revealed MRPL48 expression to be 0.930 (95% CI 0.908–0.952) in HCC and the highest cut-off value to be 4.436 TPM. In the HCCDB datasets (http://lifeome.net/database/hccdb/home.html), we also compared the transcription levels of MRPL48 between HCC and normal control sample, which further indicated that the HCC samples have abnormally high MRPL48 expression levels (Fig. 2D). Additionally, MRPL48 protein expression in HCC tissues was significantly higher than that in normal tissues, based on the CPTAC sample (https://proteomics.cancer.gov/programs/cptac) (Fig. 2E). Finally, further analysis of MRPL48 mRNA expression between HBV, HCV, and NASH (Non-alcoholic steatohepatitis)-related HCC and control samples was conducted in the GEO database, and we observed remarkably elevated MRPL48 levels in HBV- and NASH-associated HCC samples, but not in HCVs (Fig. 2F–H).

### Clinical features of HCC patients and MRPL48 mRNA levels

**Fig. 3:**
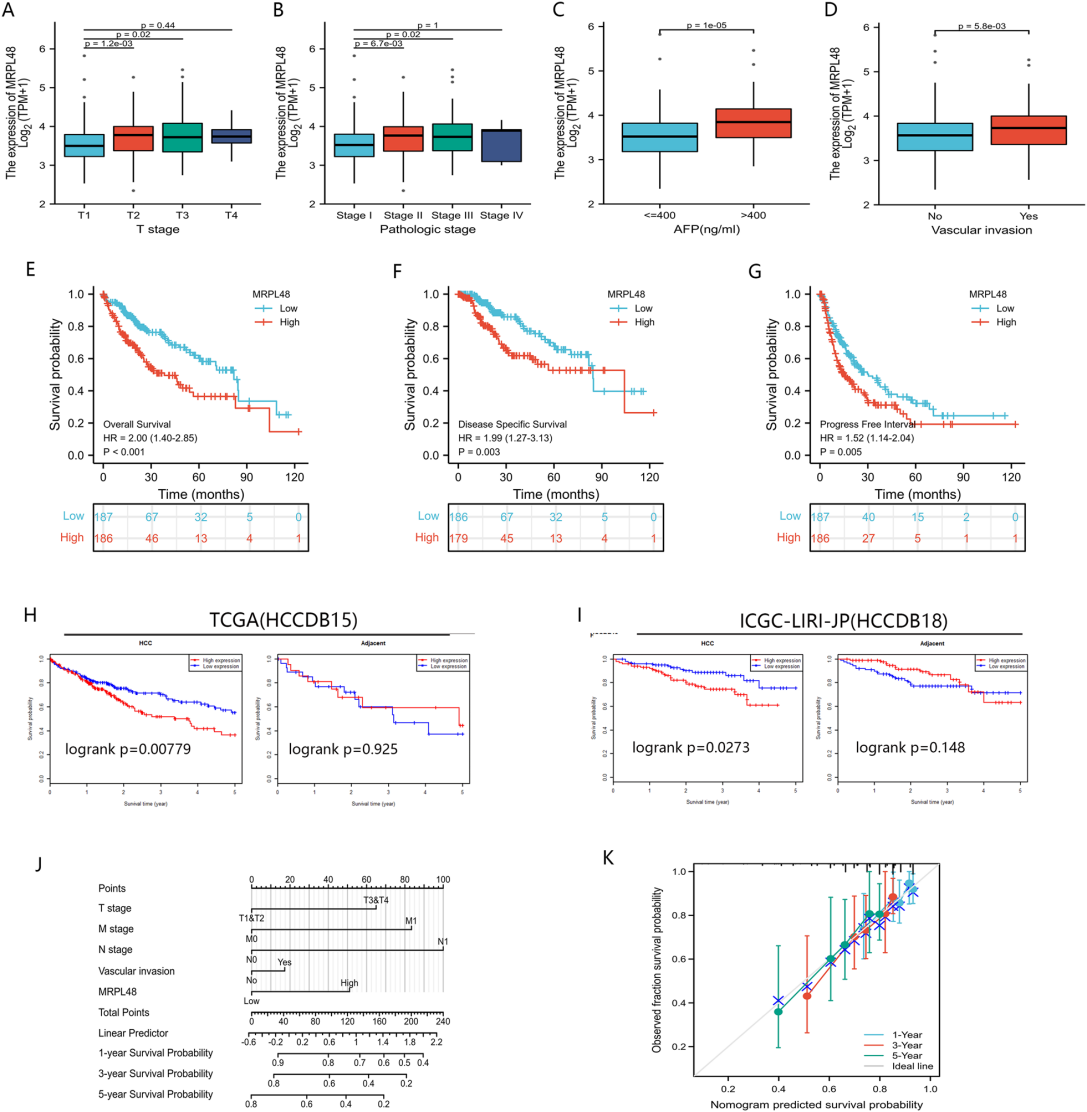
Relationship between MRPL48 expression and HCC patients' clinical characteristics and prognostic value. **A**–**D** Correlation of MRPL48 mRNA levels with T classification, the pathological tumor grade, the AFP level, and the vascular invasion of tumors of HCC patients. **E**–**G** Relationship of MRPL48 expression with OS, PFS, and PFI in TCGA datasets. **H**, **I** Association between MRPL48 expression and OS in HCCBD15 and ICGC databases. **J** A nomogram incorporating MRPL48 and other prognostic indicators from the TCGA for HCC. **K** Graph of the nomogram's calibration curve

We studied the correlation between MRPL48 expression and the clinical and pathological features of HCC patients, including the T classification, the pathological tumor grade, the AFP level, and the vascular invasion of tumors. The results indicated that higher levels of MRPL48 mRNA tended to be expressed in tissues obtained from HCC patients with advanced cancer stages (*p* < 0.01), and the highest mRNA levels of MRPL48 were predominantly found in patients at stages T2 and T3 (Fig. 3A). We also found that a significantly higher level of MRPL48 mRNA was observed in patients with high-grade tumors, and the level was significantly higher in patients at stages II and III than in patients at stage I (*p* < 0.05) (Fig. 3B). Meanwhile, increased MRPL48 mRNA expression was observed in the group with AFP greater than 400 ng/ml (*p* < 0.001) (Fig. 3C). Finally, we probably all know that invasive vascular disease often indicates that the prognosis will be worse in the future. In line with our expectations, MRPL48 mRNA expression is strongly correlated with the invasion of vessels (Fig. 3D). Additional, when MRPL48 expression was divided into low (*n* = 187) and high (*n* = 187) expression groups, its expression was significantly associated with T stage, Pathologic stage, Tumor status, Histologic grade, and AFP level (*p* < 0.05), and detailed information is shown in Table 1.

**Table 1:** Clinical characteristics correlate with MRPL48 expression

Characteristic	Low expression of MRPL48	High expression of MRPL48	*p*
*n*	187	187	
T stage, *n* (%)			< 0.001
T1	114 (30.7%)	69 (18.6%)	
T2	34 (9.2%)	61 (16.4%)	
T3	32 (8.6%)	48 (12.9%)	
T4	4 (1.1%)	9 (2.4%)	
*N* stage, *n* (%)			0.623
N0	124 (48.1%)	130 (50.4%)	
N1	1 (0.4%)	3 (1.2%)	
M stage, *n* (%)			0.628
M0	123 (45.2%)	145 (53.3%)	
M1	1 (0.4%)	3 (1.1%)	
Pathologic stage, *n* (%)			< 0.001
Stage I	105 (30%)	68 (19.4%)	
Stage II	32 (9.1%)	55 (15.7%)	
Stage III	33 (9.4%)	52 (14.9%)	
Stage IV	2 (0.6%)	3 (0.9%)	
Tumor status, *n* (%)			0.039
Tumor free	112 (31.5%)	90 (25.4%)	
With tumor	67 (18.9%)	86 (24.2%)	
Gender, *n* (%)			0.658
Female	63 (16.8%)	58 (15.5%)	
Male	124 (33.2%)	129 (34.5%)	
Race, *n* (%)			0.159
Asian	72 (19.9%)	88 (24.3%)	
Black or African American	7 (1.9%)	10 (2.8%)	
White	101 (27.9%)	84 (23.2%)	
Age, *n* (%)			0.380
< = 60	84 (22.5%)	93 (24.9%)	
> 60	103 (27.6%)	93 (24.9%)	
Residual tumor, *n* (%)			0.174
R0	172 (49.9%)	155 (44.9%)	
R1	6 (1.7%)	11 (3.2%)	
R2	0 (0%)	1 (0.3%)	
Histologic grade, *n* (%)			0.002
G1	36 (9.8%)	19 (5.1%)	
G2	96 (26%)	82 (22.2%)	
G3	47 (12.7%)	77 (20.9%)	
G4	4 (1.1%)	8 (2.2%)	
Adjacent hepatic tissue inflammation, *n* (%)			0.746
None	68 (28.7%)	50 (21.1%)	
Mild	53 (22.4%)	48 (20.3%)	
Severe	10 (4.2%)	8 (3.4%)	
AFP(ng/ml), *n* (%)			< 0.001
< = 400	127 (45.4%)	88 (31.4%)	
> 400	21 (7.5%)	44 (15.7%)	
Vascular invasion, *n* (%)			0.074
No	116 (36.5%)	92 (28.9%)	
Yes	49 (15.4%)	61 (19.2%)	
Fibrosis ishak score, *n* (%)			0.187
0	47 (21.9%)	28 (13%)	
1/2	16 (7.4%)	15 (7%)	
3/4	11 (5.1%)	17 (7.9%)	
5/6	43 (20%)	38 (17.7%)	
Age, median (IQR)	62 (53.5, 69)	60.5 (50, 68)	0.217

Survival analysis indicated that the Overall Survival (OS, defined as the period from suffering to death), Disease-Specific Survival (DSS, reflecting the death from cancer itself), and Progress-Free Intervals (PFI, from the date of randomization for initial treatment to the time of disease relapse) of HCC patients with high MRPL48 expression were significantly lower than patients with low MRPL48 expression (*p* < 0.01) (Fig. 3E–G).

According to univariate logistic regression analysis, MRPL48 expression is closely correlated with poor prognosis clinical characteristics, such as T stage (HR = 2.598, 95% CI 1.826–3.697, *p* < 0.001), tumor status (HR = 2.317, 95% CI 1.590–3.376, *p* < 0.001), pathological stage (HR = 2.090, 95% CI 1.429–3.055, *p* < 0.001), and M stage (HR = 4.077, 95% CI 1.281–12.973, *p* < 0.05) (Table 2). Aside from that, multivariate analysis also showed that MRPL48 mRNA expression is an independent prognostic factor of OS for HCC (HR = 1.729 95%, CI 1.076–2.778, *p* = 0.024). In addition, T stage (HR = 1.791, 95% CI 1.029–3.117, *p* = 0.039) and tumor status (HR = 2.317, 95% CI 1.590–3.376, *p* = 0.008) are also independent prognostic factors (Table 2) of OS.

**Table 2:** Logistic analysis of the correlation between MRPL48 expression and clinical characteristics in HCC patients

Characteristics	Total (*N*)	Univariate analysis		Multivariate analysis	
		Hazard ratio (95% CI)	*P* value	Hazard ratio (95% CI)	*P* value
T stage	370				
T1&T2	277	Reference			
T3&T4	93	2.598 (1.826–3.697)	** < 0.001**	1.791 (1.029–3.117)	**0.039**
N stage	258				
N0	254	Reference			
N1	4	2.029 (0.497–8.281)	0.324		
Tumor status	354				
Tumor free	202	Reference			
With tumor	152	2.317 (1.590–3.376)	** < 0.001**	1.882 (1.180–3.003)	**0.008**
Pathologic stage	349				
Stage I	173	Reference			
Stage III&Stage IV&Stage II	176	2.090 (1.429–3.055)	** < 0.001**	1.491 (0.812–2.738)	0.197
M stage	272				
M0	268	Reference			
M1	4	4.077 (1.281–12.973)	**0.017**	1.344 (0.319–5.658)	0.687
Residual tumor	344				
R0	326	Reference			
R1&R2	18	1.604 (0.812–3.169)	0.174		
Histologic grade	368				
G1	55	Reference			
G2&G3&G4	313	1.188 (0.721–1.958)	0.499		
AFP(ng/ml)	279				
< = 400	215	Reference			
> 400	64	1.075 (0.658–1.759)	0.772		
Vascular invasion	317				
No	208	Reference			
Yes	109	1.344 (0.887–2.035)	0.163		
MRPL48	373				
Low	187	Reference			
High	186	1.999 (1.403–2.847)	** < 0.001**	1.729 (1.076–2.778)	**0.024**

### HCC prognostic models establishment

We discovered that MRPL48 mRNA expression is an independent prognostic factor in HCC. To verify this finding, we built a model using TCGA data to predict OS based on MRPL48 mRNA expression and other clinicopathological characteristics. In our study, we integrated MRPL48 and other prognostic factors, such as the T classification, the N classification, the M classification, and the involvement of vascular structures, to construct a nomogram for OS (Fig. 3J). As a prognostic factor, a higher point on the nomogram was associated with a worse outcome. According to the calibration curve, the performance of the MRPL48 nomogram was evaluated, and the OS C index value was 0.635 (Fig. 3K). Overall, the nomogram may be more accurate than individual prognostic factors in predicting HCC survival.

### MRPL48 expression is correlated with hypomethylation in HCC

**Fig. 4:**
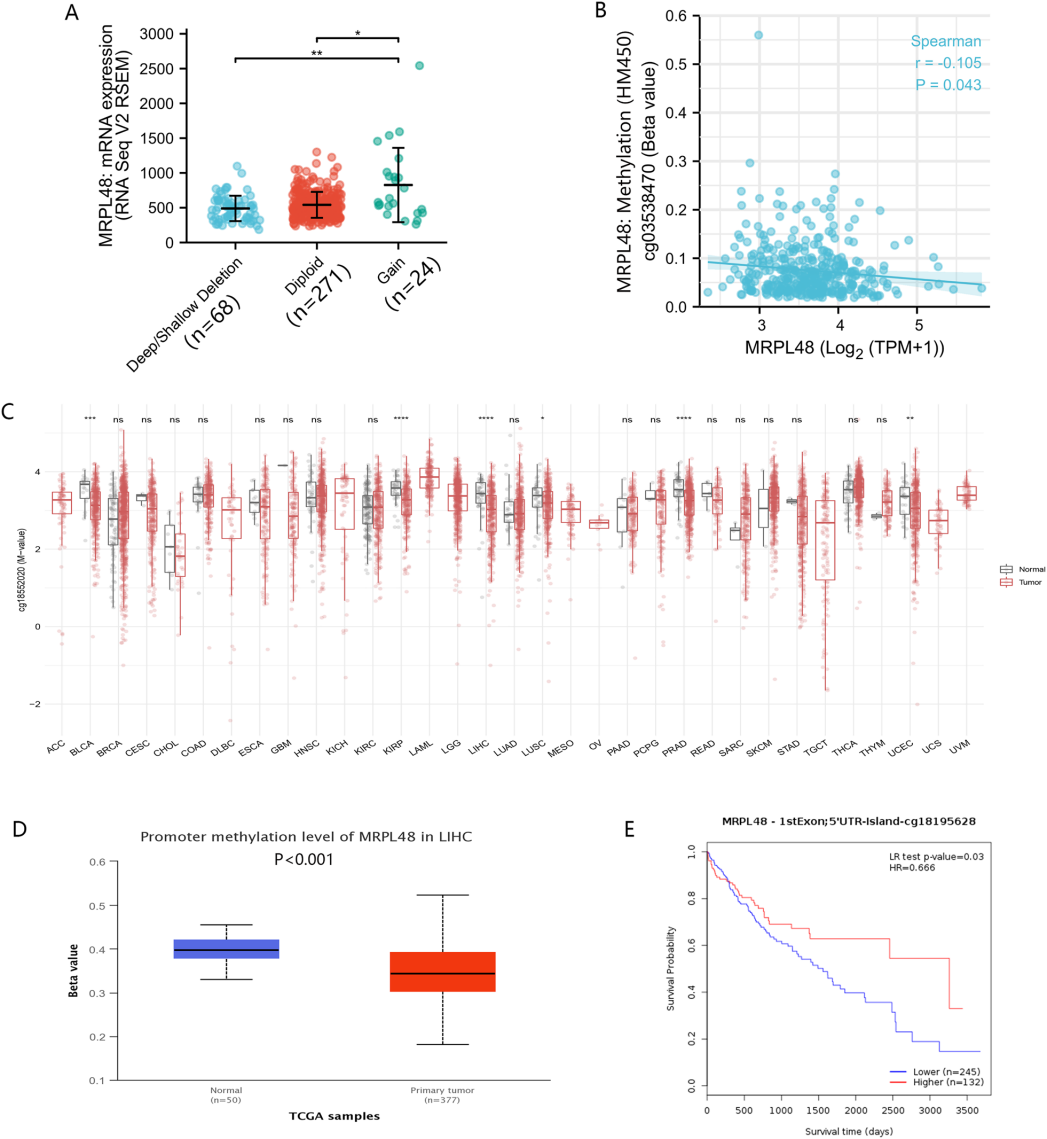
MRPL48 expression correlated with hypomethylation in HCC. **A** MRPL48 expression level in various CNV (*n* = 353). **B** The relationship between MRPL48 methylation and expression. **C** A comparison of the methylation levels of MRPL48 in pan-cancer and normal tissues from the TCGA database. **D** The promoter methylation of MRPL48 in tumor tissues (*n* = 377) and normal tissues (*n* = 50) from the TCGA HCC project. **E** The Kaplan–Meier survival analysis of MRPL48 promoter methylation in HCC (*n* = 377). *p < 0.05, **p < 0.01, ***p < 0.001, ****p < 0.0001

HCC mRNA expression was examined using cBioPortal along with copy number variation (CNV) and methylation data. The results of this study showed that only one patient with MRPL48 showed amplification of CNV. Furthermore, patients with MRPL48 CNV gain had a higher level of expression of MRPL48 in HCC, but only 6.8 percent of these patients (24/353 patients) showed this manifestation in MRPL48 (Fig. 4A). It will be interesting to see if this suggests that CNV is not a major factor inducing the high expression of MRPL48. As a result, we were able to analyze the relationship between the level of methylation of MRPL48 and the gene expression. The results from this study indicate that gene methylation is negatively correlated with MRPL48 gene expression (*R* =  − 0.105, *p* < 0.05) (Fig. 4B), and this is a promising finding. As shown in Fig. 4C, the methylation levels of MRPL48 were significantly lower than in normal tissues in many types of tumors in the TCGA database, including BLCA, KRPA, LIHC, LUSC, PRAD, and UCEC. There was a significant decrease in MRPL48 promoter methylation of TCGA HCC tumor tissues, compared with normal tissues adjacent to cancerous tissue in UALCAN (*p* < 0.001; Fig. 4D). Additionally, MethSurv analyses revealed that low MRPL48 methylation was correlated with worse OS than high methylation (p < 0.05; Fig. 4E).

### MRPL48 expression enrichment analysis

**Fig. 5:**
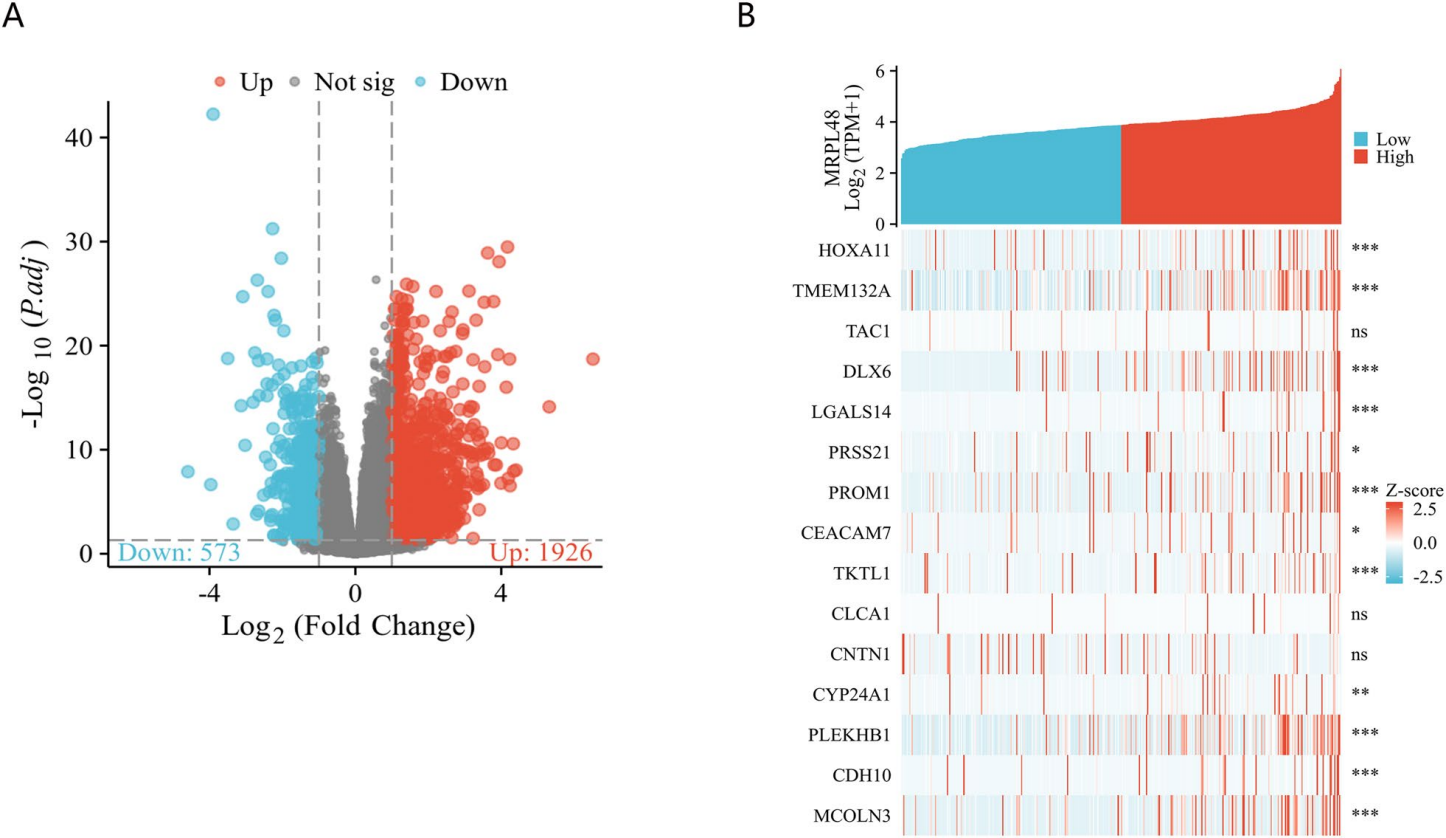
**A** Volcano Plot of differentially expressed genes (DEGs) (|log2-fold change|> 1.0 and adjusted P < 0.05). **B** Heatmap of 15 significant differentially expressed genes (DEGs) (|log2-fold change|> 1.5 and adjusted *p* < 0.05). *p < 0.05, **p < 0.01, ***p < 0.001; the “ns” indicates that the data are not significant

**Fig. 6:**
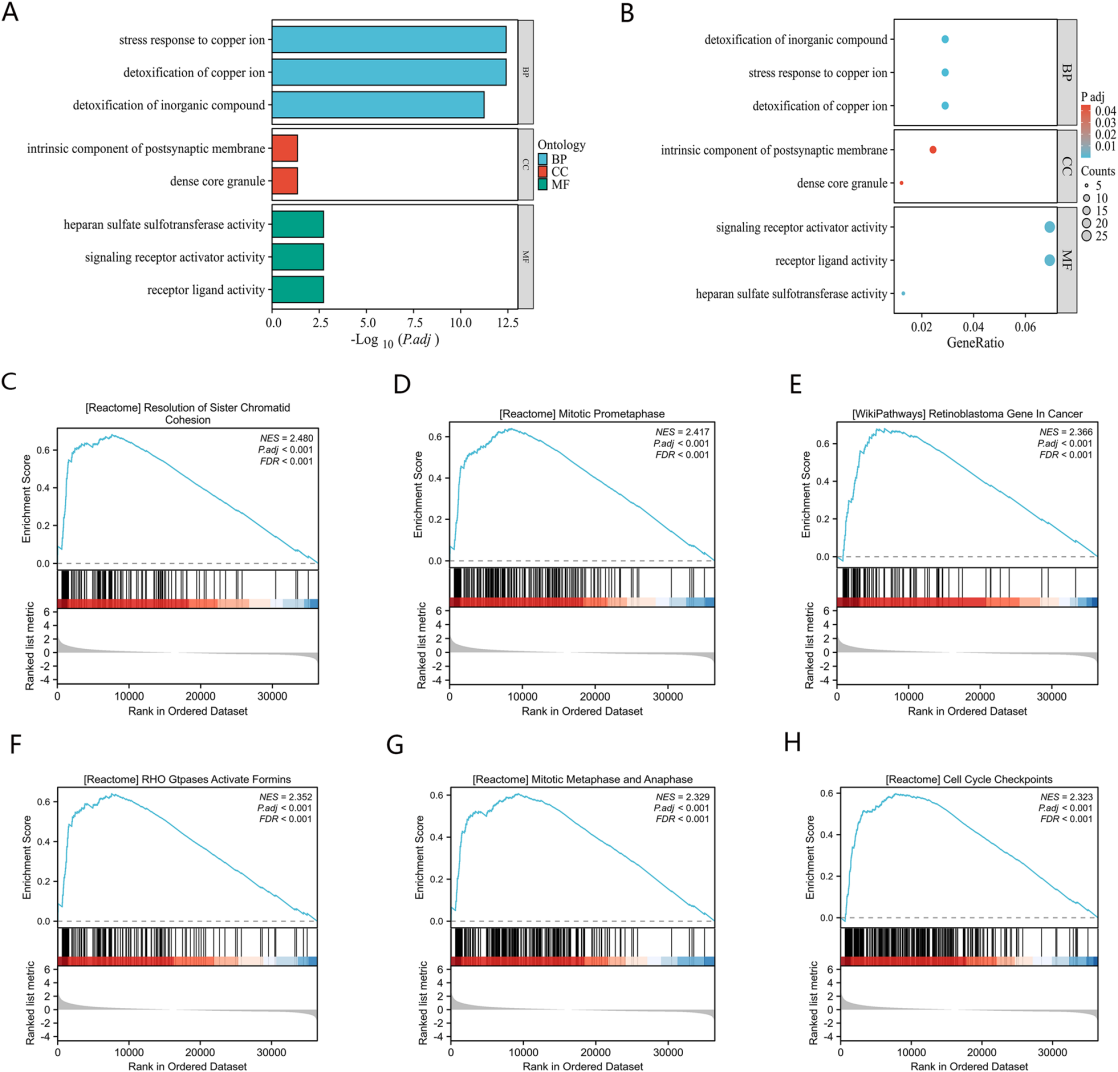
Functional enrichment of MRPL48 in HCC. **A**, **B** GO and KEGG enrichment analysis of differentially expressed genes (DEGs) in high and low MRPL48 expression samples. **C**–**H** Gene set enrichment plots of (**C**) Resolution of Sister Chromatid Cohesion, **D** Mitotic Prometaphase, **E** Retinoblastoma Gene in Cancer, **F** RHO Gtpases Activate Formins, **G** Mitotic Metaphase and Anaphase, and (**H**) Cell Cycle Checkpoints with high MRPL48 expression from GSEA

We analyzed the DEGs in tumors with high and low expression levels of MRPL48 to investigate potential mechanisms of MRPL48-mediated tumor progression. It was found that there were 2499 DEGs, 1926 of which were upregulated and 573 were downregulated. Figure 5A and B show a heatmap and volcano plot depicting DEG expression. Following this, GO enrichment analysis was used to predict the co-expression functions of the HCC patients. The top GO enrichment items in the biological processes (BP), cellular components (CC), and molecular functions (MF) groups were stress response to copper ion, detoxification of copper ion, detoxification of inorganic compound, receptor ligand activity, intrinsic component of postsynaptic membrane, dense core granule, heparan sulfate sulfotransferase activity, signaling receptor activator activity, and receptor ligand activity (Fig. 6A, B). As part of our study, we also investigated the potential pathways that lead to MRPL48 overexpression using GSEA analysis. The significantly enriched pathways included Resolution of Sister Chromatid Cohesion (Fig. 6C), Mitotic Prometaphase (Fig. 6D), Retinoblastoma Gene in Cancer (Fig. 6E), RHO Gtpases Activate Formins (Fig. 6F), Mitotic Metaphase and Anaphase (Fig. 6G), and Cell Cycle Checkpoints (Fig. 6H).

### The correlation between MRPL48 expression and immune cell infiltration

**Fig. 7:**
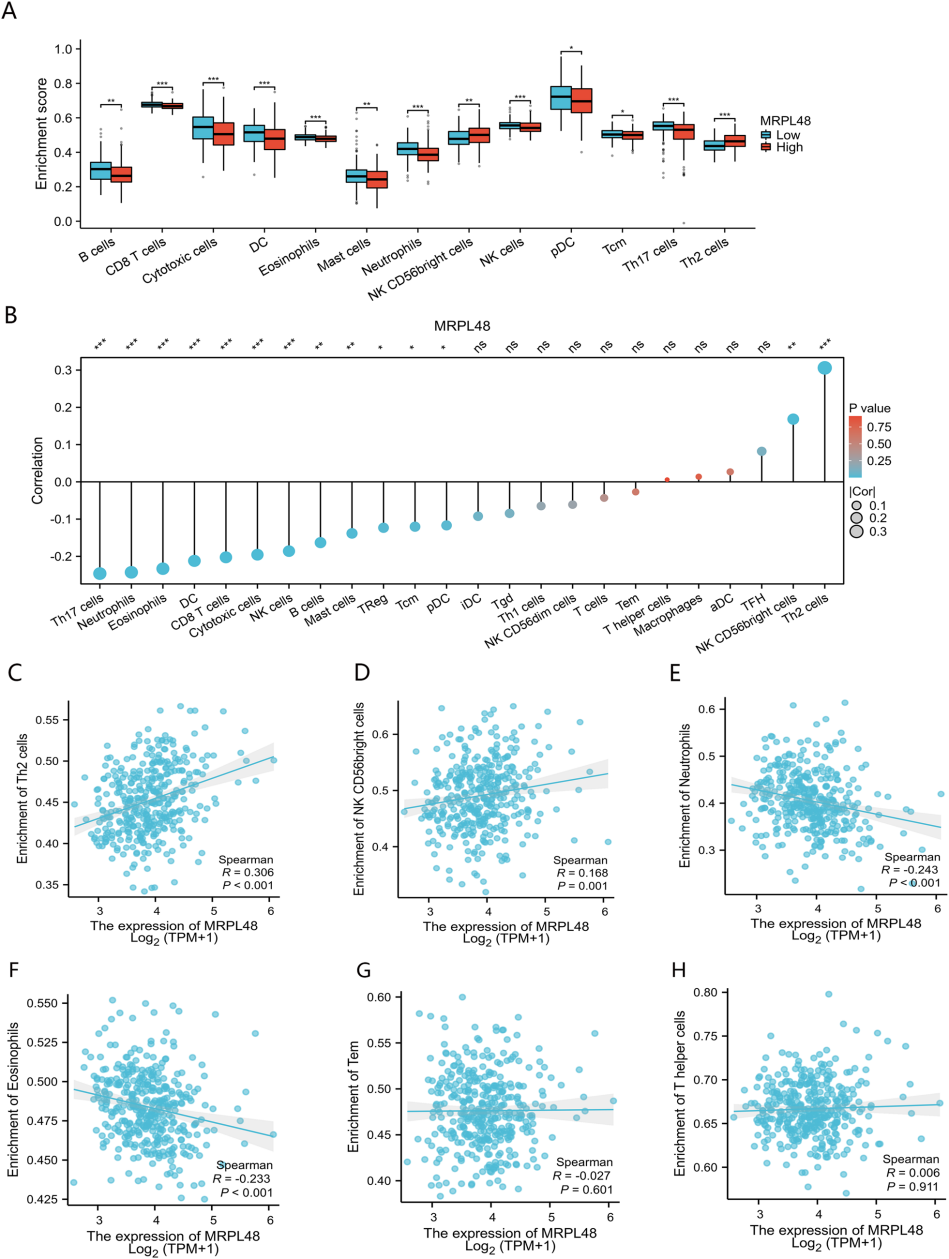
MRPL48 ssGSEA analysis and correlation with immune infiltration levels in HCC. **A** Different proportions of immune cell subtypes in HCC samples in MRPL48-high and MRPL48-low groups. **B** The correlation between the infiltration of immune cells and the expression of MRPL48. **C**, **D** The expression of MRPL48 strongly positively relates to infiltrating levels of Th2 cells (**C**), and NK CD56bright cells (**D**). **E**, **F** The expression of MRPL48 strongly negatively relates to infiltrating levels of Neutrophils (E), and Eosinophils (**F**). **G**, **H** The expression of MRPL48 is not correlated with the level of Tem (**F**) and T helper cells (H). *p < 0.05, **p < 0.01, ***p < 0.001

The biological behavior of tumors is greatly affected by the immune cells living in their microenvironment [24, 25]. Based on our investigation of the infiltration of various immune cells into the microenvironment of HCC, we found that the enrichment scores of B cells, CD8 T cells, DC, Cytotoxic cells, Eosinophils, Mast cells, Neutrophils, NK CD56bright cells, NK cells, pDC, Tcm, Th17 cells, and Th2 cells in the high MRPL48 expression group were significantly different than that in the low MRPL48 expression group (Fig. 7A). Further, we applied ssGSEA to determine whether MRPL48 mRNA expression is correlated with immune cell infiltration levels. As shown in Fig. 7B, the infiltration of immune cells was correlated with the expression of MRPL48 mRNA. The results indicate that MRPL48 mRNA expression is positively correlated with the infiltration of Th2 cells (*R* = 0.306, *p* < 0.001, Fig. 7C) and NK CD56bright cells (*R* = 0.168, *p* < 0.001, Fig. 7D). Moreover, ssGSEA showed that Neutrophils (*R* =  − 0.243, *p* < 0.001, Fig. 7E) and Eosinophils (*R* =  − 0.233, *p* < 0.001, Fig. 7F) were negatively correlated with MRPL48 expression, while Tem (*R* =  − 0.027, *p* = 0.601) and T helper cells (*R* = 0.006, *p* = 0.911) did not show a similar correlation (Fig. 7G, H). As a consequence, our study revealed a positive correlation between MRPL48 mRNA expression and NK CD56bright cells and Th2 cells.

### MRPL48 knockdown inhibited HCC cell tumorigenicity

**Fig. 8:**
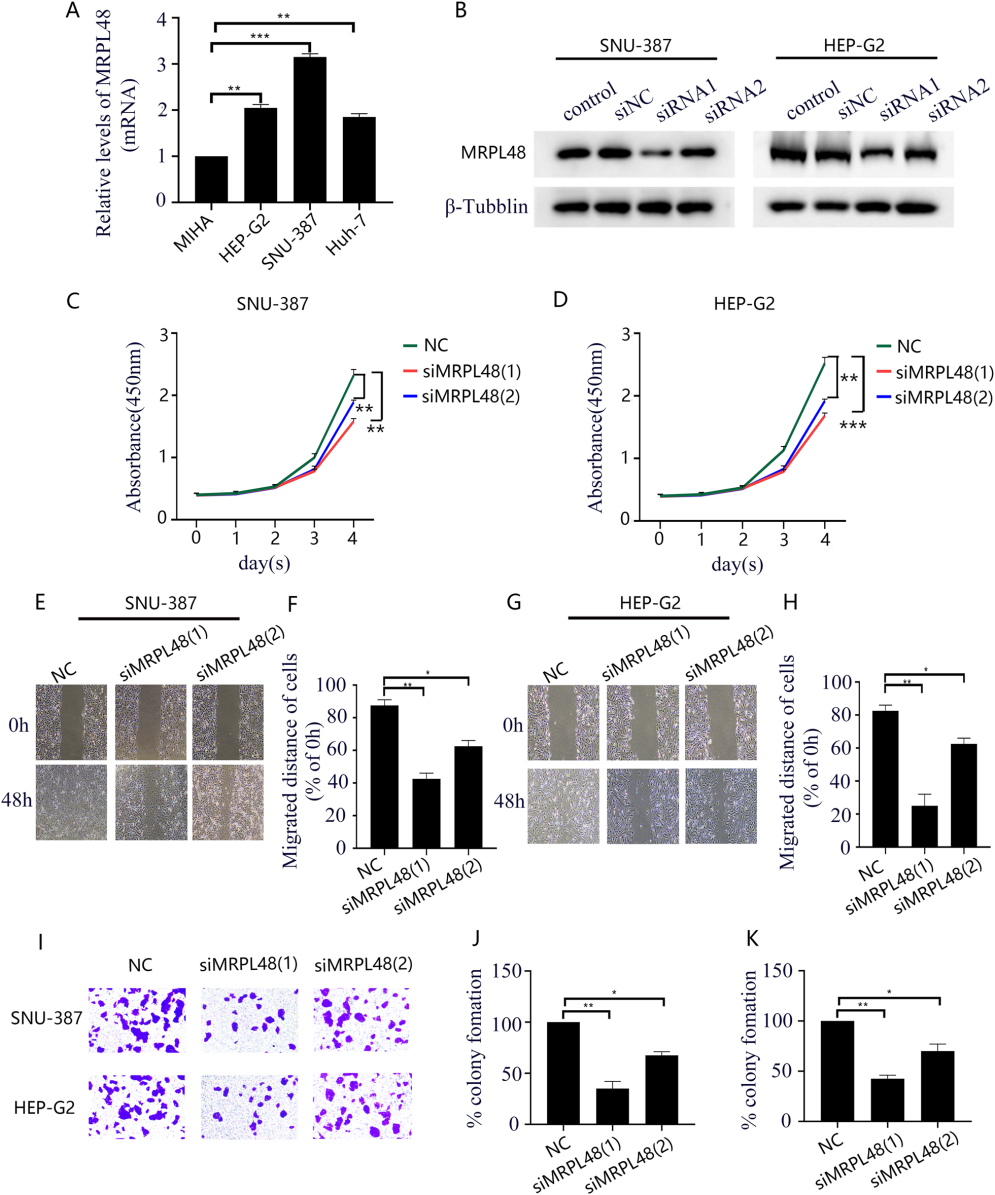
The knockdown of MRPL48 inhibits HCC cells' proliferation, migration, and invasion. **A** qRT-PCR analysis of MRPL48 expression in immortalized liver cell line and HCC cell lines. **B** Western blot analysis of the expression of MRPL48 after knockdown in SNU-387 and Hep-G2 cell lines. **C**, **D** MRPL48 knockdown affected cell viability at 24, 48, 72, and 96 h after seeding in plates by CCK-8 assay. **E**–**H** Wound healing assay analysis reflected migration ability. **I** HCC cells with MRPL48 knockdown in colony formation assay. **J**, **K** Relative quantification of colony areas is presented. Scale bars in (**I**) equal 5 mm. The data are as follows: means ± SD of three independent experiments. *p < 0.05, **p < 0.01, ***p < 0.001

The expression of MRPL48 in immortalized liver and three HCC cell lines were validated using qRT-PCR. The results showed that MRPL48 expression at mRNA levels were higher in Hep-G2, SNU-387, and Huh-7 cells, compared with MIHA cells (Fig. 8A). MRPL48 was further investigated in vitro as a potential therapeutic target for HCC. Based on the high levels of MRPL48 expression observed in the Hep-G2 and SNU-387 cell lines, these two cell lines were selected for functional analysis. The knockdown efficiency of MRPL48 was detected Western blotting (Fig. 8B). Based on the results of the CCK-8, wound healing assay, and colony formation assays, it can be concluded that MRPL48 knockdown results in the significant inhibition of the proliferation migration, and invasion abilities of Hep-G2 and SNU-387 cells (Fig. 8C–K).

## Discussion

During recent years, methods of surveillance and diagnosis of HCC have evolved. Novel biomarkers have been identified and existing surveillance tools have been repurposed [26]. However, the prevention and treatment of HCC remains a challenge in China. According to the latest estimates, the death rate due to HCC reached 4.12 million, accounting for 49.6% of the global total, respectively, in China in 2022 [27]. China has become the country with the highest incidence of HCC and the largest number of related deaths [27]. As far as HCC is concerned, it is crucial that it is detected and diagnosed as early as possible. Relevant studies have shown that the 5-year survival rate after radical surgery for small HCC (D≤5 cm) can reach 60–70% [28], while the 5-year survival rate for overall HCC is 18.4% [29]. Therefore, identifying markers that can predict and diagnose HCC occurrence and progression as early as possible has been a main focus.

The genetic and metabolic basis of most cancers are being unraveled by advances in tumor and cancer cell genomics and proteomics. Several studies indicated that cancer cells rely heavily on mitochondrial OXPHOS to provide energy, and mitochondrial ribosomes are crucial regulators of the OXPHOS system [6]. Therefore, mitochondria ribosomal biogenesis has been a critical cellular process involved during the progression of neoplastic transformation. Some progress has been achieved in determining the role of MRPS in HCC occurrence and development. It has been shown that the high levels of MRPL13 promote invasion of liver cancer cells [17]. A study by Tang found that MRPL9 is of a positive prognostic value for HCC and that the knockdown of MRPL9 and SMG5 significantly inhibits cell proliferation and migration in HCC [30]. Some researchers have found that high MRPS23 levels can predict a poor outcome in HCC, and this protein plays an important role in tumor progression [31]. Others have demonstrated that MRPS31 loss in conjunction with the upregulation of COL1A1/DDR can be used to develop a diagnostic marker for HCC [32]. In contrast, MRPL48 does not appear to have been previously reported on in HCC.

As the first step, we determined MRPL48 transcription levels in different types of cancer based on independent datasets of different sources (TCGA and GTEx, respectively). These research findings show that MRPL48 is highly expressed in a wide range of tumors. The expression of MRPL48 has been reported to be elevated in several types of cancer, including osteosarcomas and colorectal cancers. Zhang [18] used the WGC-NA algorithm and LASSO, PPI, and MOCDE methods to verify that high MRPL48 expression is associated with the poor prognosis of osteosarcoma patients. HuTT [19] reported that knockdown of MRPL48 expression using the CRISPR-Cas 9 technique could significantly increase the sensitivity of colorectal cancer cells to cetuximab. According to these findings, MRPL48 may be a factor involved in tumorigenesis. Secondly, we found that the HCC specimens had significantly high levels of MRPL48 transcription and protein, compared with the normal tissue samples. According to the current study, HCC samples from a variety of databases including HBV and NASH-related HCC samples showed higher transcriptional levels of MRPL48 than non-cancerous specimens. As was known all, hepatitis B, non-alcoholic fatty liver disease, and cirrhosis were considered as an important risk factor for the development of HCC. These findings favor our follow-up with more targeted studies. Meanwhile, a significant difference was also found between the expression of MRPL48 protein in the HCC tissues and that of normal liver tissues, as analyzed using the CPTAC database. As a follow-up to our bioinformatics analysis, we performed qRT-PCR to quantify the relative MRPL48 expression levels and our results are consistent with our bioinformatics analysis.

Furthermore, we examined the association between MRPL48 expression and clinical characteristics of patients with HCC and determined that MRPL48 expression was correlated with T classification, pathological tumor grade, AFP level, and vascular invasion. As determined by the Kaplan–Meier test, patients with HCC with high MRPL48 expression displayed unfavorable OS, DSS, and PFI prognosis. A multivariate and univariate regression analysis showed that the elevated expression of MRPL48 is an independent adverse prognostic factor in HCC. Currently, MRPL48 expression has not been reported in a prediction profile for HCC. However, our ROC curves indicate that MRPL48 expression is significant in predicting HCC. In addition, we integrated various clinical parameters into the TCGA dataset to construct a nomogram, which could be used to predict the mortality risk of individual patients and optimize their treatment. In addition, we studied the mechanisms of MRPL48 mRNA overexpression in HCC, and the results of our study suggest that MRPL48 mRNA overexpression may be associated with MRPL48 hypomethylation. Interestingly, the methylation of MRPL48 negatively affects the prognosis of HCC. HCC patients with hypomethylation have a shorter OS, which is consistent with the mRNA expression of this gene being associated with poorer survival. In spite of the fact that a wide variety of mechanisms can cause elevated gene expression, hypomethylation is one of the most important factors that contribute to it.

For a deeper understanding of how MRPL48 expression leads to abnormal changes in the downstream pathways in HCC, we analyzed DEGs in the high- and low-expressing MRPL48 in HCC patients. In the GO and KEGG enrichment analyses, the above DEGs were mostly involved in receptor ligand activity, signaling receptor activator activity, and copper metabolism. The results of the GSEA analysis indicated that the DEGs exhibited significant enrichment in mitosis, Cell Cycle Checkpoints, and cancer pathways. It has been found that all the pathways enriched above are significantly associated with the occurrence and progression of malignant tumors. A growing body of research has suggested that immune cell infiltration influences cancer development and progression [33]. In turn, this adversely affects the effectiveness of immunotherapy and the prognosis of patients. Moreover, another important finding of this study is that MRPL48 mRNA levels are associated with immune cell infiltration in HCC. MRPL48 expression is significantly correlated with the number of NK CD56 bright cells, and especially Th2 cells. Normally, the Th1/Th2 ratio of the body maintains a dynamic balance between Th1 cells and Th2 cells [34]. The release of Th2 cytokines in malignant tumors results in a Th1/Th2 imbalance caused by the Th1/Th2 drift [35]. The Th1/Th2 balance is tipped in many tumors, such as lung cancer, liver cancer, and gastric cancer, and is frequently led by Th2 dominance in the body. As a result, this may be an indication that the tumor has escaped the immune system [36]. In keeping with the above mentioned information, we observed that transcript levels of MRPL48 showed a significant correlation with Th2 cell infiltration in HCC.

Additionally, we assessed the effects of MRPL48 expression on the malignant phenotype of HCC cells in vitro. The knockdown of MRPL48 dramatically impaired Hep-G2 and SNU-387 cell proliferation, migration, and invasion. Therefore, these findings demonstrate that MRPL48 may play a crucial role in facilitating HCC cell development.

This study concluded that MRPL48 plays a significant role in HCC occurrence and development, but with some limitations. First, MRPL48 functional assessments were conducted based on an in vitro model and were not confirmed in vivo, which needs to be further explored in future studies. Second, MRPL48 expression and its prognostic value ought to be evaluated in clinical samples, since the use of publicly available datasets may lead to some inaccuracies. Finally, despite showing that MRPL48 regulates the cell cycle and influences immune infiltration, the exact biological mechanisms and signaling networks involved have not been investigated. We will perform future research to uncover the mechanism of action of MRPL48 expression in HCC.

## Conclusions

In conclusion, we systematically assessed the expression patterns, prognostic, and diagnostic value, and potential mechanisms of MRPL48 expression in the occurrence of and development of HCC in a comprehensive manner. Our results provide novel insights for identifying prognostic biomarkers and therapeutic targets. The presence of these markers may help clinicians develop better treatment plans for patients with HCC and predict their survival.

## Data Availability

Datasets from publicly available sources were used to perform the statistical analysis. Detailed information on these datasets can be obtained at https://portal.gdc.cancer.gov/ (The Cancer Genome Atlas (TCGA) program).
